# The role of bovine colostrum in feeding intolerance in preterm neonates: a systematic review and meta-analysis of randomized controlled trials

**DOI:** 10.3389/fnut.2025.1668500

**Published:** 2026-02-20

**Authors:** Jia Yao, Bo Zheng, Jie Zhang, Fen Xu, Jianbo Zhuang

**Affiliations:** 1 NICU, Suzhou Ninth People's Hospital, Suzhou, China; 2 Global Visiting Department, UNC of Greensboro, Greensboro, NC, United States

**Keywords:** feeding intolerance, bovine colostrum, preterm neonate, NEC, TFF120, systematic review, meta-analysis

## Abstract

**Background:**

Feeding intolerance is common in preterm infants and may lead to poor weight gain and prolonged hospitalization. Bovine colostrum, owing to its rich content of bioactive components such as immunoglobulins, lactoferrin, and growth factors, has been suggested to reduce feeding intolerance and promote gut maturation in preterm infants; however, available clinical trials provide inconsistent evidence regarding its efficacy. Therefore, this study aimed to conduct a systematic review and meta-analysis to evaluate the effects of bovine colostrum supplementation on feeding intolerance, necrotizing enterocolitis, and time to full enteral feeding in preterm infants.

**Method:**

We searched five databases for randomized controlled trials (RCTs) on bovine colostrum (BC) use in preterm neonates from their inception to 8 July 2025. Outcomes included feeding intolerance, necrotizing enterocolitis (NEC), and time to full enteral feeding to 120 mL/kg/d (TFF120). Data were analyzed using RevMan 5.3.

**Result:**

A total of four RCTs (670 infants) were included in this study. BC supplementation was associated with a lower incidence of feeding intolerance (RR = 0.76; 95% CI: 0.61–0.94) with low heterogeneity (*I*^2^ = 2%). However, this effect should be interpreted with caution, as the lack of blinding in the included trials may have introduced performance bias. No significant effects were observed for NEC or TFF120, and the evidence regarding adverse events was limited.

**Conclusion:**

BC cannot be recommended for preterm infants based on current evidence. The observed reduction in feeding intolerance is likely due to performance bias, with no proven benefit for NEC or TFF120.

**Systematic review registration:**

https://www.crd.york.ac.uk/PROSPERO/view/CRD420251102646, identifier CRD420251102646.

## 1 Introduction

Infants—particularly those born very preterm (gestational age <32 weeks) and/or with very low birth weight (VLBW, <1,500 g)—frequently experience feeding difficulties due to the immaturity of their gastrointestinal system, including delayed motility, limited digestive capacity, and underdeveloped immune function.

Maternal milk (MM) is widely regarded as the optimal nutritional source for very preterm infants (VPIs), as it has been consistently associated with a lower incidence of feeding intolerance compared to donor human milk or preterm formula (PF) (1). In contrast, infant formula (IF) is comparatively less effective in promoting gastrointestinal maturation, enhancing feeding tolerance, reducing NEC, and supporting optimal neurodevelopment in preterm infants (2). However, MM alone is often insufficient to meet the nutritional demands of VLBW infants; therefore, human milk fortification (HMF) is routinely required to optimize growth and neurodevelopment, including in low- and middle-income countries. When mother’s own milk (MOM) is unavailable, preterm infant formula has long been shown to provide benefits in growth and neurodevelopment. In addition, donor milk (DM) may be suboptimal due to the reduced bioactivity of pasteurized and thawed human milk, which is typically collected from women during the later stages of lactation (3).

BC is a nutrient-dense fluid that contains a high concentration of protein (80–150 g/L) and is enriched with a wide array of bioactive compounds, including lysozyme, immunoglobulins, transforming growth factors (TGFs), insulin-like growth factors (IGFs), and epidermal growth factors (EGFs). These components are believed to play essential roles in gut maturation, immune modulation, and tissue repair, making BC a promising nutritional supplement for preterm infants (4, 5). In preterm pigs, commonly used as a translational model for preterm infants, exclusive or partial enteral feeding with BC has been shown to reduce the incidence of NEC and promote postnatal gastrointestinal development and function (6, 7). The safety, tolerability, and feasibility of BC feeding in preterm neonates have been investigated in pilot clinical trials conducted in Denmark and China (8). BC supplementation has been associated with a reduced time to achieve TFF compared to exclusive PF, particularly in clinical settings characterized by slow feeding advancement and limited availability of MOM (8). However, no meta-analysis has yet systematically evaluated the impact of BC supplementation on feeding intolerance, NEC, and the achievement of TFF. Therefore, we conducted a systematic review and meta-analysis to assess the effects of bovine colostrum on feeding intolerance. Statistical analyses were also performed for NEC and the achievement of TFF120.

Beyond its nutritional value, bovine colostrum exerts multiple biological effects that may underlie its clinical benefits in preterm infants. Its bioactive components—such as immunoglobulins, lactoferrin, lysozymes, and various growth factors—have been shown to strengthen mucosal barrier integrity, enhance epithelial cell proliferation, and modulate intestinal inflammation. These components may also promote colonization by beneficial microbiota and inhibit pathogenic bacteria through antimicrobial peptides and immunomodulatory cytokines (9).

## 2 Methods

### 2.1 Eligibility criteria

The Preferred Reporting Items for Systematic Reviews and Meta-Analyses (PRISMA) guidelines were followed as a checklist for this study, and the study protocol has been registered with PROSPERO (Registration ID: CRD420251102646), available at: https://www.crd.york.ac.uk/PROSPERO/view/CRD420251102646. The inclusion criteria, based on the population, intervention, comparison, outcomes, and study (PICOS) framework, were as follows: the study population comprised preterm infants receiving enteral feeding support. The intervention group received breast milk or formula supplemented with bovine colostrum powder, while the control group received breast milk or formula alone. The primary outcome was the incidence of feed intolerance. Feeding intolerance was defined across the included trials as the presence of gastrointestinal symptoms such as large gastric residuals, abdominal distension, vomiting, or bloody stools that required reduction, interruption, or cessation of enteral feeding. The secondary outcomes included the incidence of NEC and TFF120. Only randomized controlled trials (RCTs) with accessible full-text publications were included, with no language restrictions.

The exclusion criteria were as follows: (1) observational studies, including cohort, case–control, and cross-sectional studies or case series; (2) unpublished abstracts; and (3) non-primary research such as reviews, editorials, or commentaries.

### 2.2 Search strategy

A systematic literature search was conducted across five electronic databases: EMBASE, PubMed, the Cochrane Library, Web of Science, and Scopus. The search was last updated on 8 July 2025.

In PubMed, the following search terms were used: ((((feed intolerance [Title/Abstract]) OR (feeding intolerance [Title/Abstract])) OR (enteral feeding [Title/Abstract])) AND (randomized [Title/Abstract])) AND (bovine colostrum [Title/Abstract]). In addition, the reference lists of all included studies were manually screened to identify additional relevant publications. The complete search strategies for all databases are detailed in the Supplementary material.

### 2.3 Study selection

Study selection was performed independently by two authors (JY and BZ), who screened the titles, abstracts, and full texts for eligibility. Any discrepancies were resolved through discussion and, if necessary, by consultation with a third author (JieZ).

### 2.4 Data extraction

Extracted study details included the first author, year of publication, sample size, study population, basic demographic and clinical characteristics (e.g., age, weight, sex, and APGAR scores), and reported outcomes. All data were independently extracted by two reviewers. Any discrepancies were resolved through discussion or, when necessary, by consulting a third reviewer. When essential data were not available in the published articles, the corresponding authors were contacted for additional information or clarification.

### 2.5 Study risk of bias assessment

Two authors used Review Manager 5.4 (10) to assess the risk of bias from all studies, such as random sequence generation, allocation concealment, blinding, incomplete outcome data, selective reporting, and other sources of bias. The assessment results were classified into high, low, or unclear.

### 2.6 Data synthesis

Review Manager (RevMan) version 5.4 (Cochrane Collaboration) and Stata/MP version 14.0 were used to perform the meta-analysis. For continuous outcomes, the mean difference (MD) and corresponding 95% confidence interval (CI) were calculated using the inverse variance method. For dichotomous outcomes, the risk ratio (RR) with 95% CI was calculated. A random-effects model was used when substantial heterogeneity was detected; otherwise, a fixed-effects model was used. Heterogeneity was assessed using the *I*^2^ statistic and interpreted as low (*I*^2^ < 25%), moderate (*I*^2^ = 26–50%), or high (*I*^2^ > 50%) (11). The results were considered significant if the two-tailed *p*-value was ≤0.05.

### 2.7 Assessment of publication bias

An assessment of publication bias was not performed in this study, as funnel plot analysis typically requires a minimum of 10 studies to achieve adequate power for detecting asymmetry. Nonetheless, it is important to acknowledge that the findings may still be subject to publication bias due to the limited number of the included studies (10).

### 2.8 GRADE

The Grading of Recommendations Assessments, Development and Evaluation (GRADE) analysis was used to evaluate the quality of evidence and was completed (12).

## 3 Results

### 3.1 Study selection

**Figure 1:**
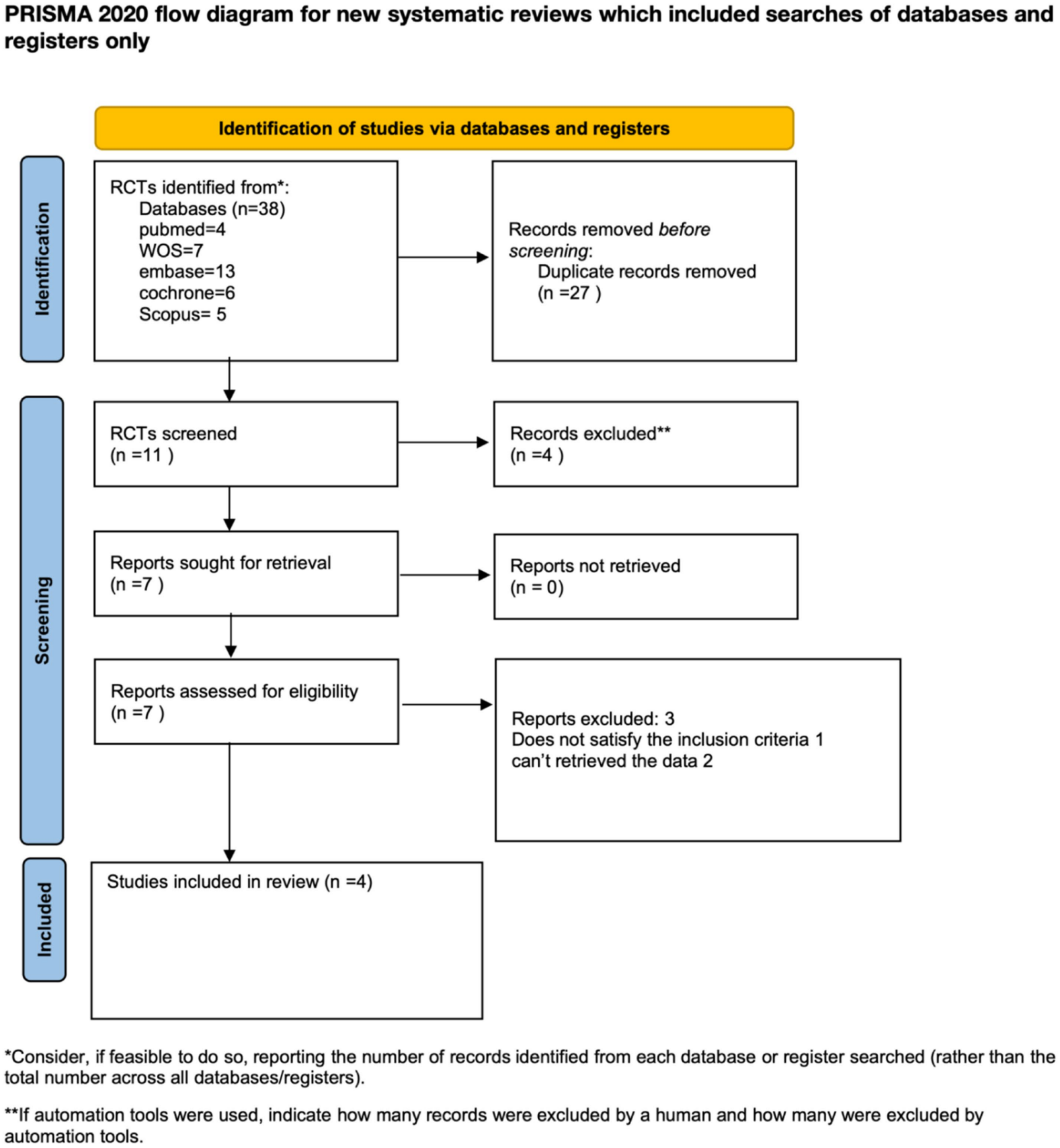
PRISMA flow diagram. PRISMA, Preferred Reporting Items for Systematic Reviews and Meta-Analyses. Adapted from: Page MJ, McKenzie JE, Bossuyt PM, Boutron I, Hoffmann TC, Mulrow CD, et al. The PRISMA 2020 statement: an updated guideline for reporting systematic reviews. BMJ 2021;372:n71. doi: 10.1136/bmj.n71.

A total of 38 articles were retrieved in the initial search from the databases. Among them, 27 articles were removed as duplicates. Three studies were then excluded after a full-text review. Ultimately, four records were included in this systematic review. A detailed PRISMA flow diagram for the study selection process is shown in Figure 1.

### 3.2 Characteristics of the included studies

Two studies were conducted in Egypt (13, 14), and two studies were conducted in China (8, 15). The minimum and maximum number of participants was 40 (16) and 350 (15), respectively. All studies included preterm infants. The control group in two studies received human milk (HM), including maternal milk and donor milk (8, 15). The control group in three studies received formula, including infant formula and preterm formula (13–15). Two studies specified the time points for assessing feeding intolerance, with evaluations conducted once during the first and second weeks, respectively (8, 15). The remaining two studies did not report the timing of assessment (13, 14). Among the four randomized controlled trials included in this meta-analysis, three studies (8, 14, 15) involved maternal milk feeding in either the intervention or control groups, whereas one study (13) used exclusively formula feeding without maternal milk. In the meta-analysis of feeding intolerance, the outcome measured at 2 weeks was selected over the 1-week assessment. This decision was made to capture a more stable and representative evaluation of feeding tolerance, as early transient symptoms in the first week may not accurately reflect the overall feeding outcome. One study reported both intention-to-treat (ITT) and per-protocol (PP) analyses (15). The ITT analysis was chosen for inclusion in this review over the per-protocol (PP) analysis to preserve the benefits of randomization and minimize the risk of bias introduced by non-random attrition or protocol deviations (for details, refer to Table 1). For missing data, we contacted the authors of trial reports or extracted data from figures using the WebPlotDigitizer tool. The extracted data and included studies’ characteristics are shown in Table 1. Since this study is a meta-analysis, the table only presents research findings included in at least two studies. For example, studies on bovine colostrum side effects such as cow milk protein allergy were not included, as only one study (15) provided relevant data.

**Table 1:** Effect of bovine colostrum.

Study ID	Year	Country	Group	*N*	Birth weight (g), mean ± SD	Birth GA (weeks), mean ± SD	Male (*n*)	Final dose for BC (g/kg/day)	Time of initiation	Method of colostrum administration	Outcomes	Feed intolerance	NEC (stages II and III)	TFF120	Randomization method
Yan et al. (15)	2023	China	HM + PF + BC	171	1336.93 ± 280.81	29.87 ± 1.37	107	Unclear	14d	Oropharyngeal	Multiply	41 (156)	5 (148)	28 ± 17.77	Randomly permuted blocks
			HM + PF	179	1346.28 ± 266.22	29.75 ± 1.41	104		14d	Oropharyngeal	Multiply	60 (167)	4 (154)	27 ± 16.29	
Ismail et al. (14)	2021	Egypt	PF + BC	32	1562.63 ± 341.66	32.6 ± 1.31	15	0.91 g/kg/day	14d	Oropharyngeal	Multiply	1	0	-	Computer-generated randomization sequence
			PF	48	1494.13 ± 313.65	32.18 ± 1.58	24		14d	Oropharyngeal	Multiply	9	5	-	
Juhl et al. (8)	2018	China+ Danish	HM + BC	21	1,560 ± 104	30.7 ± 0.68	13	1 mL/kg/day	14d	Oropharyngeal	Multiply	3 (10)	-	10.8 ± 5.91	Randomly permuted blocks
			HM	19	1,406 ± 131	30.2 ± 0.84	10		14d	Oropharyngeal	Multiply	4 (10)	-	14.9 ± 17.2	
Farag et al. (13)	2024	Egypt	IF+BC	100	1,184 ± 211	30 ± 1	55	4.5 g/kg/day	14d	Oropharyngeal	Multiply	42	0	-	Randomly permuted blocks
			IF	100	1,231 ± 216	31 ± 1	53		14d	Oropharyngeal	Multiply	48	2	-	

NEC, necrotizing enterocolitis; GA, gestational age; BC, bovine colostrum; TFF, full enteral feeding; HM, human milk; PF, preterm formula; IF, infant formula.

Two trials (8, 15) used ColoDan/BC powder provided by Biofiber-Damino (Denmark), one study (14) used Immuguard® (Dulex-Lab, Egypt), and another study (13) used Baby-Steps (NIG Nutritionals, New Zealand). The sources and trade names of bovine colostrum used in the included studies are provided in Table 2.

**Table 2:** Sources, trade names, and manufacturers of bovine colostrum used in the included clinical trials.

Study year	Products/trade names	Manufacturers	Country of origin	Notes
Juhl et al. (8)	ColoDan	Biofiber-Damino	Gesten, Denmark	Intact, unmodified bovine colostrum powder; reconstituted for enteral feeding
Yan et al. (15)	BC powder	Biofiber-Damino	Gesten, Denmark	Gentle low-temperature pasteurization, gamma irradiation, and spray-drying
Farag et al. (13)	Baby-Steps	NIG Nutritionals	Auckland, New Zealand	LDS used to enhance bioavailability

BC, bovine colostrum; LDS, liposomal delivery system.

The definition of feeding intolerance varied among the trials: in the PreColos RCT (15), it was defined as the withholding of at least one feed or having excessive gastric residuals; in the bovine colostrum pilot trial (8), it was defined as gastric residuals with vomiting or abdominal distension requiring feed interruption; in the Egyptian ROP study (13), it was defined as bilious vomiting or residuals >50% of the feed volume; and in another study (14), it was defined as ≥3 days of persistent symptoms such as vomiting, distension, and residuals.

### 3.3 Risk of bias assessment

**Figure 2:**
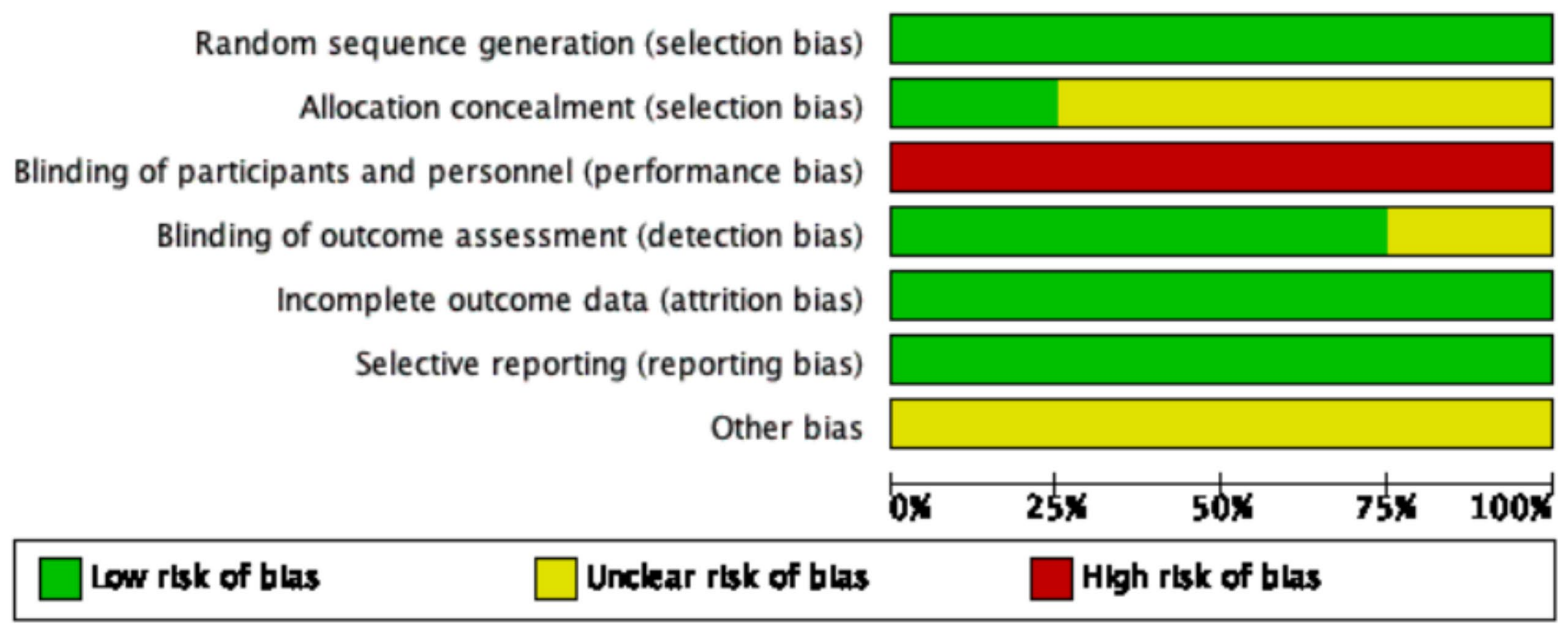
Graph for the risk of bias.

**Figure 3:**
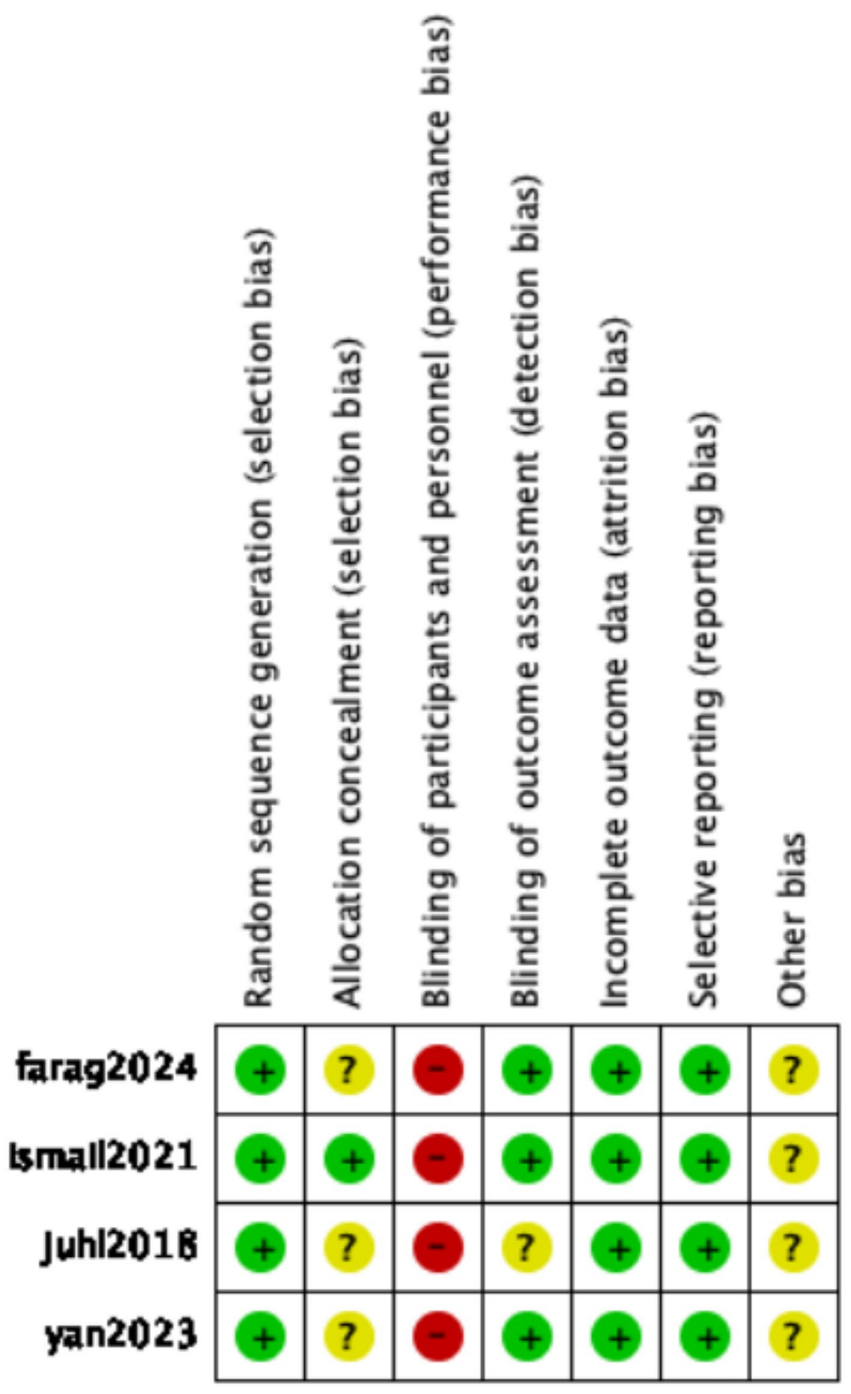
Summary of the risk of bias.

The visual representation of bias assessment is shown as a risk of bias graph (Figure 2) and table (Figure 3). In the risk of bias assessment, the domain “blinding of participants and personnel” was judged to be at high risk due to the inability to implement blinding, as bovine colostrum differs in color from standard milk or formula. The original study authors stated that blinding was not feasible due to the different appearance of bovine colostrum compared with standard formula or human milk. However, alternative approaches, such as coloring or masking the formula, could have been implemented to minimize this difference. Therefore, the absence of blinding should be regarded as a major methodological limitation that may have introduced performance bias and potentially exaggerated the observed effects on feeding intolerance. All studies used randomization, and the method of randomization was clearly specified.

### 3.4 Primary outcomes

#### 3.4.1 Feeding intolerance

**Figure 4:**
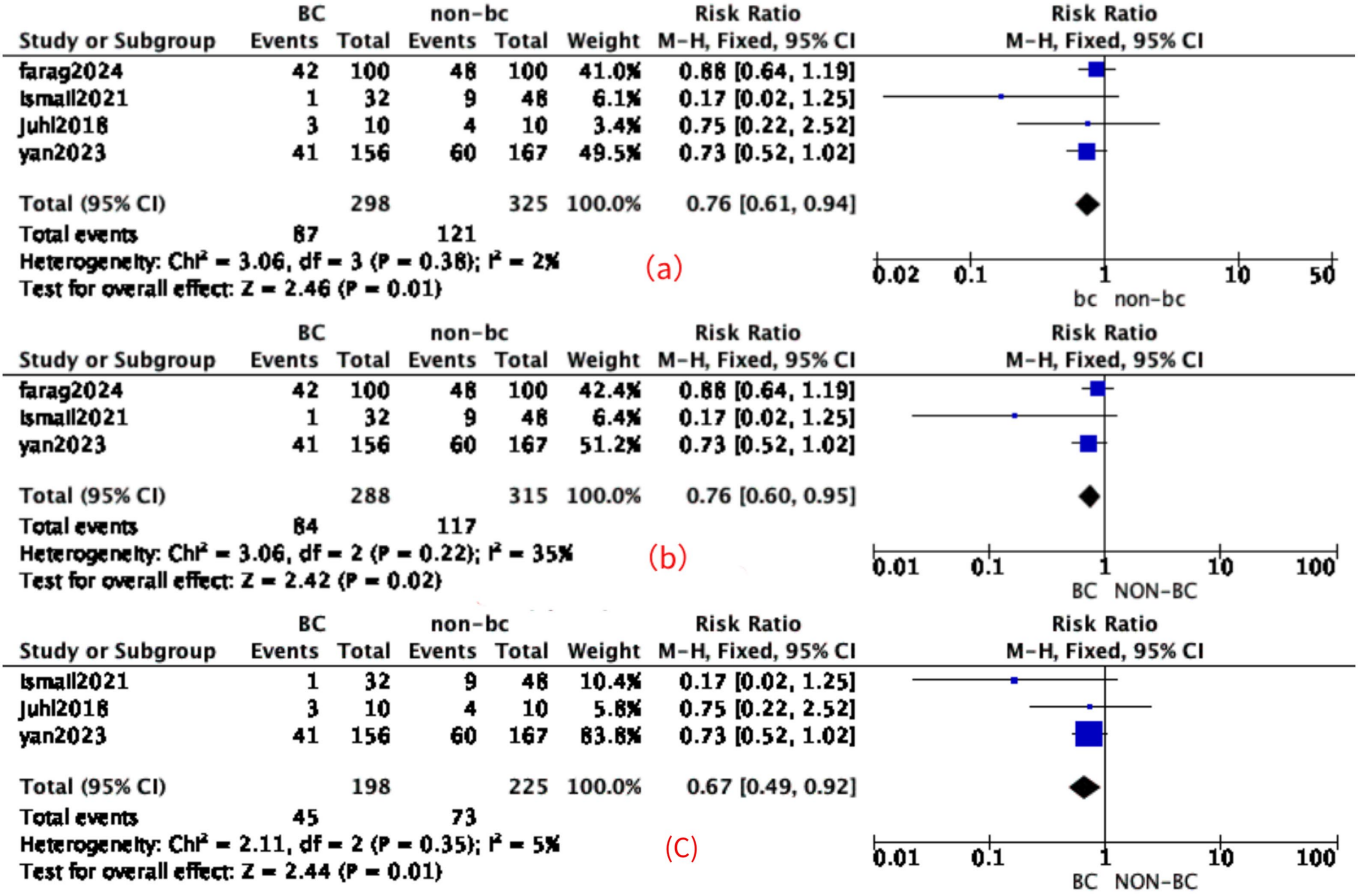
Effect of BC in feeding intolerance. **(a)** Forest plot of the effect of BC in feeding intolerance. **(b)** Subgroup analysis evaluating the effect of bovine colostrum on feeding intolerance, with studies of small sample size (*n* < 50) excluded. **(c)** Subgroup analysis, excluding the study in which no maternal milk feeding was provided.

A total of four included studies (*n* = 670) reported feeding intolerance. The pooled analysis showed that a decrease in the incidence of feeding intolerance compared to the control group was associated with the BC and control combination group [RR: 0.76 (95% CI: 0.61, 0.94), *p* < 0.01] with low heterogeneity, *I*^2^ = 2% (moderate grade level). In the subgroup analysis, studies with a sample size of fewer than 50 participants were excluded. The results remained significant [RR: 0.76 (95% CI: 0.60, 0.95), *p* = 0.02] with moderate heterogeneity (*I*^2^ = 35%). In another subgroup analysis, the data were obtained after excluding the study in which no maternal milk feeding was provided; the results remained significant [RR: 0.67 (95% CI: 0.49, 0.92), *p* = 0.01] with low heterogeneity (*I*^2^ = 5%). Studies with the detail are shown in Figure 4. Due to the low heterogeneity, sensitivity analysis was not conducted.

### 3.5 Secondary outcomes

#### 3.5.1 NEC

**Figure 5:**
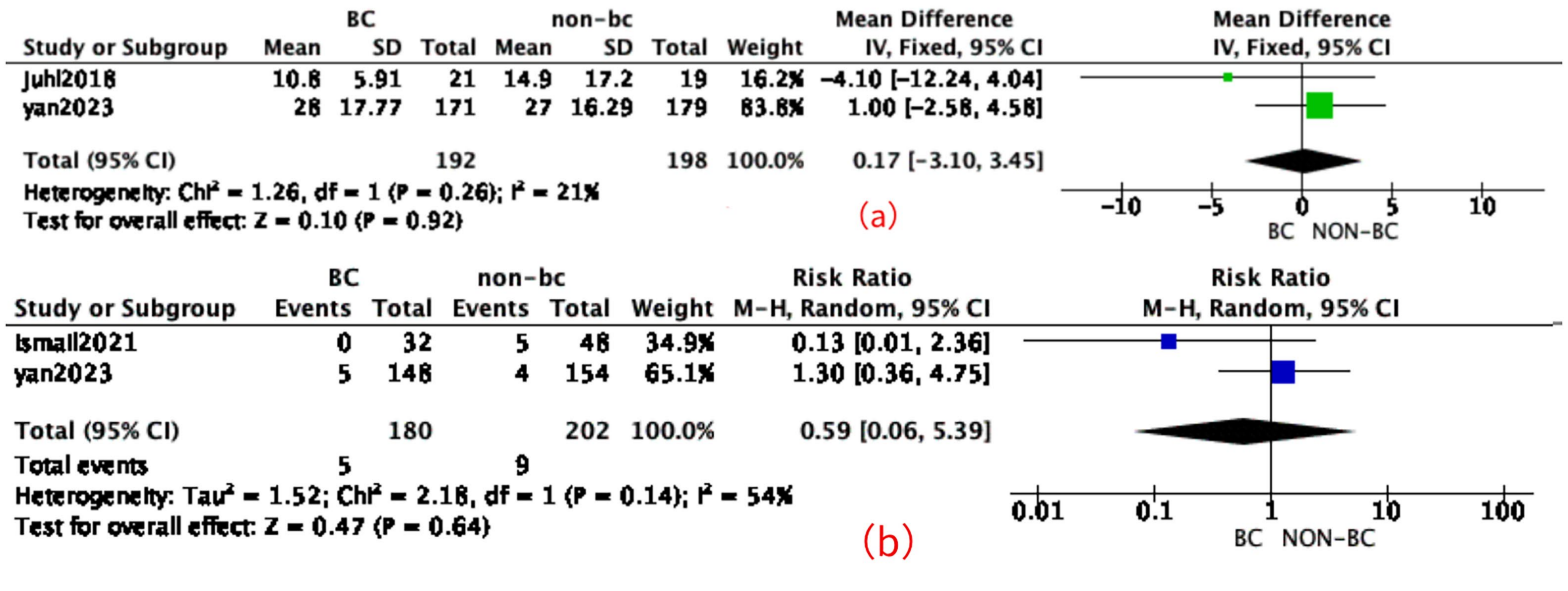
Effect of BC in NEC and TFF120. **(a)** Forest plot for BC in TFF120. **(b)** Forest plot for BC in NEC.

Three included studies (*n* = 582) reported the incidence of NEC after intervention. In one study (13), inconsistencies were identified regarding the incidence of NEC: while the main text of the article reported a higher NEC rate in the BC group, the Supplementary material presented the opposite. Due to this discrepancy, the NEC outcome from this study was not included in the quantitative synthesis, and readers should be aware of this limitation. The statistical analysis did not demonstrate a significant difference compared to the control group (RR = 0.59, 95% CI: 0.06–5.39, *p* = 0.64), with high heterogeneity observed, *I*^2^ = 54% (very low grade level); the detail is shown in Figure 5.

#### 3.5.2 TTF120

Two included studies (*n* = 390) reported TTF120. The statistical analysis did not demonstrate a significant difference compared to the control group [MD 0.17 (95% CI -3.1, 3.45), *p* = 0.92, fixed-effects model], with a low degree of heterogeneity, *I*^2^ = 21% (low grade level); the detail is shown in Figure 5. Due to the limited number of included studies and the low heterogeneity, sensitivity and subgroup analyses were not conducted.

### 3.6 The GRADE of this study

A GRADE assessment of the meta-analyses outcomes for the objective showed that there is a moderate to very low certainty of evidence for these results, as shown in Table 3. Two outcomes were downgraded for imprecision due to wide confidence intervals, one outcome was downgraded for moderate heterogeneity, and all outcomes were downgraded for potential publication bias, as the limited number of the included studies made it difficult to rule out its presence.

**Table 3:** GRADE level.

Grade level for bovine colostrum for feeding intolerance	Study limitation	Imprecision	Inconsistency	Indirectness	Publication bias	Grade
Feeding intolerance	No downgrade	No downgrade	No downgrade	No downgrade	Downgraded due to the limited number of included studies.	Moderate
NEC	No downgrade	Downgrade due to wide confidence intervals	Downgrade due to *I*^2^ = 33%	No downgrade	Downgraded due to the limited number of included studies.	Very low
TFF120	No downgrade	Downgrade due to wide confidence intervals	No downgrade	No downgrade	Downgraded due to the limited number of included studies.	Low

## 4 Discussion

This meta-analysis was conducted to evaluate the efficacy of BC in managing feeding intolerance, NEC, and time to TFF120 in preterm neonates. Four studies met the inclusion criteria. The results showed a reduction in the incidence of feeding intolerance; however, no statistically significant effects were observed for NEC or TFF120.

Bovine colostrum has been shown to promote gut maturation by enhancing intestinal cell proliferation and differentiation. It also improves the microscopic structure of the intestine, likely by acting as a growth factor for enterocytes (17, 18). This effect may be attributed to its content of various growth factors, including insulin-like growth factor (IGF), which has been reported to enhance feeding tolerance (8, 19). The timing of bovine colostrum administration was consistent across the included studies, with all initiating supplementation on day 14 of life. However, the dosage and target volume varied among the studies. In addition, the type of base nutrition differed between the groups: some experimental groups received a combination of human milk and formula, while others were provided with only one nutrition. These variations may have contributed to clinical and methodological heterogeneity, potentially influencing the overall statistical outcomes.

Although bovine colostrum (BC) contains bioactive components beneficial for gut development, our study did not demonstrate a preventive effect against necrotizing enterocolitis (NEC), which is consistent with the findings of previously published meta-analyses (20). This may be attributed to the extreme immaturity of the preterm gut, potential immunological reactions to bovine proteins, and the multifactorial pathogenesis of NEC. Moreover, variations in dosage and timing of administration, as well as the absence of adjunctive interventions, may have limited the efficacy of BC alone in preventing NEC. The contradictory reporting of NEC incidence in one study (13) further limits the strength of the available evidence and underscores the need for rigorous and transparent reporting in future trials.

In this meta-analysis, BC supplementation did not produce a statistically significant effect on the time to TFF. Several factors may explain this finding. First, differences in baseline feeding protocols—such as the rate of feeding advancement and the use of maternal milk versus formula—could have confounded the observed effects of BC. Second, some included studies had small sample sizes or administered BC only for a short duration, which may have limited its impact on long-term feeding outcomes. Third, variations in the timing and dosage of BC supplementation across studies may have reduced comparability and weakened the statistical power of the pooled analysis.

An important aspect that warrants further discussion is the striking difference between the effect of BC on feeding intolerance and its lack of benefit on the time to achieve TFF120. While our pooled analysis, in line with previous reports, suggested that BC supplementation may reduce the incidence of feeding intolerance, this effect did not translate into a shorter time to reach full enteral feeding. This discrepancy was also clearly observed in the largest trial conducted to date, the Chinese study (15), which reported a considerable reduction in feeding intolerance but no improvement—and even a negative trend—in the time to reach TFF120. These findings suggest that the observed benefit on feeding tolerance may, at least in part, be attributable to performance bias in the absence of blinding, rather than a true physiological effect on gastrointestinal maturation. Therefore, future rigorously designed, double-blinded randomized controlled trials are needed to clarify whether BC can genuinely accelerate feeding progression in preterm infants.

Furthermore, it should be noted that recent large-scale trials, including the Chinese study (15), and the Danish pilot trial (8), reported inconsistent findings. Both studies suggested a reduction in feeding intolerance, but neither demonstrated a significant benefit for NEC or time to full feeds defined as 120 mL/kg/day. Importantly, the absence of double blinding in these trials may have introduced performance bias, potentially exaggerating the observed effects on feeding intolerance. In addition, the Danish study reported a higher incidence of metabolic acidosis and showed that BC did not perform better than standard human milk fortifier. These findings highlight that, despite promising early signals, the clinical efficacy of BC remains uncertain and requires further validation in rigorously designed, double-blinded randomized controlled trials.

## 5 Limitations

First, due to the distinct color difference between bovine colostrum and breast milk or formula, double blinding was not feasible in the majority of studies, potentially introducing performance or detection bias. Second, the small number of available studies increases the risk of publication bias and limits the generalizability of the findings. Third, the intervention and control group criteria varied across studies: some trials used only maternal or donor milk, while others included preterm formula, contributing to clinical heterogeneity. Fourth, although all studies initiated BC supplementation on day 14 of life, the specific dosage regimens and target volumes differed considerably, which may have influenced outcome comparability and introduced additional heterogeneity. Fifth, because the definition of feeding intolerance differed across studies, one would theoretically expect increased heterogeneity. Surprisingly, we instead observed low heterogeneity, suggesting possible performance bias (e.g., due to lack of blinding). Similar paradoxical findings were also seen for the association with time to achieve full enteral feeding.

Therefore, well-designed, double-blind, placebo-controlled trials with standardized feeding protocols and larger sample sizes are needed to more definitively determine the efficacy of bovine colostrum in preterm infants.

## 6 Conclusion

Based on the present evidence, feeding BC to preterm infants cannot be recommended. Although a considerable effect was observed on feeding intolerance, this is most likely attributable to performance bias resulting from the lack of blinding in the included trials. No significant benefits were found for NEC or time to TFF120. Further high-quality, adequately powered, and double-blinded randomized controlled trials are required to clarify the potential role of BC in preterm infant nutrition.

## Data Availability

The original contributions presented in the study are included in the article/Supplementary material, further inquiries can be directed to the corresponding author.
