# Supplementary material for: The role of bovine colostrum in feeding intolerance in preterm neonates: a systematic review and meta-analysis of randomized controlled trials
Source: Front Nutr. 2026 Feb 20;12:1668500. doi: 10.3389/fnut.2025.1668500 (PMC12962896; doi:10.3389/fnut.2025.1668500)
Supplement: Supplementary file 1 [file Data_Sheet_1.PDF]

Search nu	Query	Sort By	Filters	Search De	Results	Time	Date
5	((intoleranc	Most Recent		"intoleranc	4	9:58:49	7/8/2025
4	intolerance	Most Recent		"intoleranc	45,214	9:58:40	7/8/2025
3	randomize	Most Recent		"randomiz	766,651	9:58:17	7/8/2025
1	bovine col	Most Recent		"bovine cc	1,049	9:57:22	7/8/2025
